# Access to and interest in human milk research opportunities among Black pregnant and postpartum people

**DOI:** 10.3389/fnut.2024.1274833

**Published:** 2024-04-12

**Authors:** Ifeyinwa V. Asiodu, Caryl L. Gay, Brandi Gates-Burgess, Gabriela Negrete

**Affiliations:** ^1^Department of Family Health Care Nursing, School of Nursing, University of California, San Francisco, San Francisco, CA, United States; ^2^Highland Hospital, Alameda Health System, Oakland, CA, United States; ^3^Department of Human Ecology, Human Development & Family Studies, College of Agricultural and Environmental Sciences, University of California, Davis, Davis, CA, United States

**Keywords:** human milk research, lactation research, recruitment, research participation, Black women and birthing people, research opportunities

## Abstract

**Background:**

Concerns exist regarding biomedical research participation in marginalized and historically disadvantaged communities.

**Objectives:**

The purpose of this study was to understand critical barriers to participation in human milk research from the perspective of Black pregnant and postpartum people.

**Methods:**

A national sample of Black pregnant and postpartum people (*n* = 104) was recruited to complete a cross-sectional online survey informed by the Life Course Perspective. Survey questions assessed research experiences and preferences, particularly related to human milk research, knowledge of historical events/policies targeting Black communities, and demographic characteristics. A socio-economic composite score was calculated as an indicator of socio-economic advantage. Survey data were summarized descriptively and potential correlates of research engagement were evaluated.

**Results:**

Most (69%, *n* = 71) respondents reported previous participation in a research study, yet only 8 (8%) reported ever being asked to participate in a breastfeeding/chestfeeding or human milk study, and one respondent was unsure. Despite so few having been asked, 59% (*n* = 61) of respondents indicated they would donate breast/human milk to research if asked. Respondent characteristics associated with prior research participation included having greater socio-economic advantage (*p* = 0.027) and greater knowledge of discriminatory historical events/policies (*p* < 0.001). In contrast, the only respondent characteristic associated with willingness to donate human milk to research was younger age (*p* = 0.002).

**Conclusion:**

Our findings suggest that Black pregnant and postpartum people are interested in biomedical research, specifically human milk and lactation research. However, greater intentionality and targeted recruitment of this underrepresented population is needed to increase diversity among human milk and lactation study samples. Structural and community-based interventions, informed by community members, are needed to address concerns and improve participant engagement.

## Introduction

Breastfeeding and the provision of human milk have a significant impact on health for both mothers and infants across their life course (1–3). The benefits noted are short and long term (1–3). Lactating people experience rapid uterine involution and decreased bleeding, greater weight loss, and stronger dyad bonding, and also have reduced risk of depression, uterine cancer, ovarian cancer, breast cancer, cardiovascular disease, and type 2 diabetes later in life (2, 3). Breastfed infants experience fewer infections, specifically gastrointestinal, respiratory, and ear infections, and have lower rates of necrotizing enterocolitis, sudden infant death syndrome, obesity, and type 1 and type 2 diabetes (1–3). Human milk has also been shown to support infant growth and development, a healthy immune system, and gut health (4).

The current national maternal and child health focus on increasing breastfeeding and access to human milk has generated a number of initiatives geared toward investigating how the varied composition of human milk contributes to health. Until recently, human milk composition was thought to be very consistent and uniform across all populations, and that characteristics such as race, age, parity, or diet did not greatly affect milk composition (5). However, recent literature has shown that human milk is a dynamic, bioactive fluid with significant inter-individual variability (6). Human milk provides important nutrients needed for growth and development; however other bioactive components of human milk also play a significant role in shaping infant behavior and neurodevelopment (7).

Studies that include collection and analysis of human milk and other biospecimens are needed to develop a comprehensive understanding of the effect of life course factors on human milk production and composition across all populations. Yet, research shows Black women are less likely to donate biological specimens (8–10) and are underrepresented in biomedical research generally, especially in studies focused on human milk composition (9). A recent review of 28 human milk composition studies conducted from 1980 to 2016 noted that many studies did not disclose participants’ race/ethnicity and of those studies that disclosed, the majority of participants self-identified as white and healthy (11).

Concerns exist regarding biomedical research in marginalized and historically disadvantaged communities, particularly when collection of biological specimens is involved and the direct benefit to participants may be low (11). Community engagement prior to recruitment is recommended to ensure that research is conducted responsibly, ethically, and appropriately (12, 13). Reasons for lack of participation include distrust of the healthcare system and ineffective or biased recruitment, as well as unfamiliar procedures and participant burden (8–10). Further, ethical concerns, lack of awareness, economic and geographical barriers, lack of culturally informed recruitment methods, limited diversity among research team members, and previous studies such as the U.S. Public Health Service (USPHS) Syphilis Study at Tuskegee and use of HeLa cells have also been identified as suggested barriers (14). A number of these barriers signify current sociocultural factors and historical factors dating back to slavery and segregation (15, 16). While several barriers to participation in medical research, specifically related to clinical trials have been noted, few studies have reported barriers to participating in human milk research (17). The purpose of this study was to understand critical gaps to participation in human milk research from the perspective of Black pregnant and postpartum people.

## Methods

### Study design and ethics

This study was designed as part of a larger cross-sectional online survey of Black pregnant and postpartum people and healthcare workers. This analysis focuses specifically on survey data collected from the sample of Black pregnant and postpartum people; analysis of the sample of healthcare workers is reported separately. The study design was informed by the Life Course Perspective, which seeks to examine lives through a structural, social, historical, and cultural lens and provides a framework to examine how personal history and life events impact current health and future decision making (18, 19). The four key elements of the Life Course Perspective framework include: timeline, timing, environment and equity (20). The study protocol was approved by the Institutional Review Board at the University of California, San Francisco (protocol #18–24,803). All participants provided both verbal consent during the eligibility screening and online consent as part of the online survey. All data were collected between November 2020 and February 2021. All participants were compensated for their time with a $50 electronic gift card.

### Recruitment and eligibility

Convenience sampling was used to recruit study participants by posting study flyers and advertisements on community bulletin boards, public health programs, hospitals, clinics, and social media platforms (e.g., Facebook, Twitter, and Instagram). Prospective participants were given initial information about the study’s purpose, risks, and benefits by the research team. Prior to being sent the online qualitative survey by email, eligibility was confirmed by phone, and consent was obtained through the online survey. Eligibility criteria for the survey of Black pregnant or postpartum people were: (1) self-identify as Black or African American, (2) currently pregnant or parenting a child under the age of 5 years, (3) able to read and write English, (4) based in the US and (5) over 18 years of age.

### Online survey

The online survey was conducted using the Qualtrics XM platform and included questions about the participants’ demographic characteristics (e.g., age, gender identity, racial/ethnic identity, education, employment status, type of health insurance, income, state of residence), pregnancy and/or birth characteristics, and their experiences with, interest in, and preferences about research participation and engagement, specifically research focused on breastfeeding/chestfeeding and human milk. Questions about participants’ knowledge of historical events and policies targeting Black populations (e.g., Jim Crow laws, Tuskegee Syphilis experiments, J. Marion Sims’ surgical experiments on enslaved African women, and Henrietta Lacks) were also included. The term “chestfeeding” has recently been introduced to describe feeding a baby from one’s chest and is often preferred by parents who identify as transgender or non-binary (2, 3).

### Analysis

Statistical analyses of quantitative survey data were conducted using Stata Statistical Software, release 15 (StataCorp, College Station, TX). Descriptive statistics included means, standard deviations, and ranges for continuous variables, and frequencies and percentages for categorical variables. Continuous variables were checked for normality. Responses were evaluated for differences by four indicators of socio-economic advantage: (1) college degree (yes/no), employment (yes/no), (2) private health insurance (yes/no), and annual income of $50,000 or more (yes/no). A socio-economic composite score was also calculated as the number of indicators of socio-economic advantage that each respondent had (range 0–4). A knowledge composite score was calculated as the number of the four historical events and policies each respondent reported at least some knowledge of (range 0–4). Chi-square or Fisher’s exact tests were used for group comparisons on categorical variables as appropriate. Logistic regression was used to estimate odds ratios with 95% confidence intervals for associations between categorical variables. Independent-sample *t*-tests or Wilcoxon’s rank sum tests were used to compare two groups on continuous variables as appropriate. *p*-values <0.05 were considered statistically significant.

### Description of authors’ backgrounds

All authors are trained and/or experienced researchers who worked collaboratively to create the online qualitative survey. Author 1 is an experienced community-based participatory action, qualitative, and mixed methods researcher. Author 2 is an experienced quantitative researcher and data analyst. Authors 3 and 4 are experienced community researchers. Authors 1 and 3 are also experienced International Board-Certified Lactation Consultants (IBCLCS). Author 1 is a Black woman and Associate Professor in Nursing. Author 2 is a White woman, Research Specialist and Data Analyst. Author 3 is a Black woman, IBCLC and Director of a lactation focused non-profit. Author 4 is a Latinx woman and Clinical Research Coordinator. All of the authors bring their own lived experiences and understanding as it relates to the issue of access to research opportunities and interest in human milk research participation, which may affect our analysis and interpretation of the data.

## Results

### Sample characteristics

Of the 191 people who expressed interest in this study, 109 (57%) were eligible for the survey of Black pregnant and postpartum people, 51 (27%) were eligible for the survey of health care workers, 4 (2%) were not eligible for both survey, and 27 (14%) could not be reached for screening. Of the 109 people eligible for the survey of Black pregnant and postpartum people, 104 (95%) completed the online survey and were included in the analysis. Sample characteristics are summarized in Table 1. All participants identified as Black (*n* = 100) or multi-racial (*n* = 4) and all identified as women. The sample was geographically diverse, representing 23 US states.

**Table 1:** Sample characteristics (*N* = 104).

Sample characteristic	Statistics
**Pregnancy /parenting status**	
Pregnant, % (*n*)	25% (26)
Gestation in weeks, mean (SD) [range]	21 (8.8) [8–38]
Parenting a child ≤5 years old, % (*n*)	75% (78)
Age of youngest child in years, mean (SD) [range]	1.2 (1.1) [0–5]
**Age in years**	
Mean (SD)	32.1 (4.9)
Median [range]	32 [21–49]
**Education, % (*n*)**	
High school graduate or equivalent	8% (8)
Some college (1–3 years) or technical school	21% (22)
College graduate	34% (35)
Graduate school (Advanced degree)	37% (39)
**Employment status, % (*n*)**	
Employed for wages	56% (58)
Self-employed	6% (6)
Unemployed/Looking for work	9% (10)
Stay-at-home parent	22% (23)
Student	3% (3)
Unable to work	3% (3)
Prefer not to answer	1% (1)
**Health insurance, % (*n*)**	
Medicaid	31% (32)
Private insurance	60% (63)
Both medicaid and private Insurance	2% (2)
Tricare	4% (4)
Uninsured	3% (3)
**Annual income, % (*n*)**	
$0 - $25,000	17% (18)
$25,000 - $50,000	33% (34)
$50,000 - $75,000	18% (19)
$75,000 - $100,000	10% (10)
$100,000 and up	16% (17)
Prefer not to answer / missing	6% (6)
Socio-economic composite*, % (*n*)	*n* = 98*
0	9% (9)
1	20% (20)
2	18% (18)
3	22% (21)
4	31% (30)
Region of residence, % (*n*)	
Northeast	18% (19)
Midwest	11% (11)
South	35% (36)
West	36% (38)
Knowledge** of historical events and policies targeting Black populations, mean (SD) [range]	
Jim Crow laws	3.4 (1.1) [1–5]
Tuskegee Syphilis experiments	2.8 (1.2) [1–5]
J. Marion Sims’ surgical experiments on enslaved African women	2.7 (1.3) [1–5]
Henrietta Lacks and the HeLa cell line	2.6 (1.4) [1–5]

*The socio-economic composite is the count of 4 socio-economic indicators (college education, employment, private insurance, and income > $50,000/year) and excludes the 6 participants who did not report an income.

** Knowledge scores ranged from 1 = “Not at all knowledgeable” to 5 = “Most knowledgeable (expert)”.

### Research access, participation, and preferences

Table 2 summarizes the survey respondents’ prior experiences with research studies. Most of the women in this sample (69%, *n* = 71) reported having previously participated in a research study. However, only eight respondents (8%) reported ever having been asked to participate in a breastfeeding/chestfeeding or human milk research study, one respondent was unsure, and only three of these nine respondents (33%) reported having participated in such studies. Despite few having been asked, 59% (*n* = 61) of the respondents in this sample indicated that they would donate breast or human milk to a research study if asked.

**Table 2:** Research experiences, interest, and preferences (*N* = 104).

Survey question	% (*n*)
**Have you ever participated in a research study?**	
No	28% (29)
Unsure	4% (4)
Yes	69% (71)
**Have you ever been asked to participate in a breastfeeding/ chestfeeding OR human milk research study, before today?**	
No	91% (95)
Unsure	1% (1)
Yes	8% (8)
**If asked, would you donate breast or human milk to a research study?**	
No	11% (11)
Unsure	30% (31)
Yes	59% (61)
Missing	1% (1)
**Where would you prefer to be approached about such studies? (Check all that apply) [*listed in order of frequency*]**	
At a healthcare setting	84% (87)
At a community setting	75% (78)
At a religious setting	20% (21)
Other setting: social media	13% (13)
Other setting: anywhere, stores, library, prenatal yoga, childcare centers, breastfeeding classes, non-profit, word of mouth from family/friends, somewhere I can ask real questions and get real answers	11% (11)
Other setting: online or virtually	6% (6)
Other setting: email, text	3% (3)
**How would you prefer to learn about breastfeeding/ chestfeeding or human milk studies you might be eligible for? (Check all that apply) [*listed in order of frequency*]**	
By sending an email about the study	79% (82)
By advertising the study on social media (please specify which ones): Instagram, Facebook, Twitter, Reddit, What to Expect, all platforms	62% (64)
In person	55% (57)
By sending a text message about the study	42% (44)
By advertising the study on a website (please specify which ones): hospital, university, or library websites, any website serving the needs of postpartum parents, Baby Center, ROSE, WebMD	16% (17)
By advertising the study on a smartphone app (please specify which ones): Facebook, Instagram, Twitter, Snapchat, What to Expect, The Bump, Peanut, Ovia, Flo, Glow, pregnancy and parenting apps	14% (15)
Some other way (please specify): podcasts, mailings, flyers, parenting magazines, doctor’s office, radio, news, trusted community organizations/leaders, word of mouth	8% (8)
**Who would you prefer to approach you about participating in breastfeeding/chestfeeding or human milk studies? (Check all that apply) [*listed in order of frequency*]**	
A lactation support person	88% (91)
A doula	78% (81)
My healthcare provider	75% (78)
A community health worker	67% (70)
A member of the research team	63% (65)
A public health nurse	62% (64)
Someone else: someone I know/trust, family or friends, another mother/breastfeeding mom, Midwife, WIC staff, community leader	12% (12)

Table 2 also summarizes respondents’ preferences about where, how, and by whom they want to be approached about research studies related to breastfeeding/chestfeeding or human milk. A majority of respondents preferred to be approached about such studies in a healthcare (84%) or community (75%) setting, and preferred recruitment methods were to receive an email about the study (79%), see an advertisement about the study on social media (62%), or learn about it in person (55%). Most respondents were open to learning about breastfeeding/chestfeeding and human milk studies from a range of people, including a lactation support person (88%), doula (78%), or their health care provider (75%).

Table 3 summarizes the experiences of the nine respondents who were or may have been recruited for a prior breastfeeding/chestfeeding or human milk research study. Although the numbers are small, few respondents reported participating in such studies, receiving a breast pump or other supplies as part of a study requesting a human milk sample, or that the research team was diverse or consisted of a Person of Color. Most of these nine respondents (67%) reported receiving strong support from their support system during the time they were approached to participate in a breastfeeding/chestfeeding or human milk research study. For those who were recruited but did not participate in a breastfeeding/chestfeeding or human milk research study, respondents were split as to whether having a different type of support system would have motivated them to participate.

**Table 3:** Breastfeeding/chestfeeding and human milk research experiences among those who were or may have been recruited for such studies (*n* = 9).

Survey questions to those who answered “Yes” or “Unsure” to whether they had ever been asked to participate in a breastfeeding/chestfeeding OR human milk research study before today	% (*n*)
**Have you ever participated in a breastfeeding/chestfeeding research study (focused on your experiences and behaviors) before today?**	
No	56% (5)
Unsure	11% (1)
Yes	33% (3)
[*If “Yes” or “Unsure”*]	
**Was the research team diverse or consist of a person of color?**	*n* = 4
No	25% (1)
Unsure	50% (2)
Yes	25% (1)
**Have you ever participated in a human milk research study (team asked for breast/human milk sample) before today?**	
No	67% (6)
Unsure	–
Yes	33% (3)
[*If “Yes” or “Unsure”*]	
**Did the research team provide you with a breast pump and other supplies?**	*n* = 3
No	67% (2)
Yes	33% (1)
**Was the research team diverse or consist of a Person of Color?**	*n* = 3
No	33% (1)
Unsure	33% (1)
Yes	33% (1)
**What type of support system did you have during the time you were approached to participate in a breastfeeding/ chestfeeding OR human milk research study?**	
Strong support	67% (6)
Moderate support	–
Neutral support	22% (2)
No support	–
Negative support	–
Unsure	11% (1)
[*If “Yes” or “Unsure” if they ever participated in a breastfeeding/chestfeeding OR human milk research study*]	
**How supportive was your support system supportive of your decision to participate in the breastfeeding/chestfeeding or human milk study?**	*n* = 5
Very supportive	20% (1)
Somewhat supportive	20% (1)
Somewhat unsupportive	40% (2)
Very unsupportive	–
Unsure	20% (1)
[*If “No” or “Unsure” if they ever participated in a breastfeeding/chestfeeding OR human milk research study*]	
**Would having a different type of support system have motivated you to participate in a breastfeeding/chestfeeding or human milk research study?**	*n* = 7
Definitely yes	–
Probably yes	14% (1)
Probably not	29% (2)
Definitely not	14% (1)
Unsure	43% (3)

### Knowledge of historical events and policies targeting Black populations

**Figure 1:**
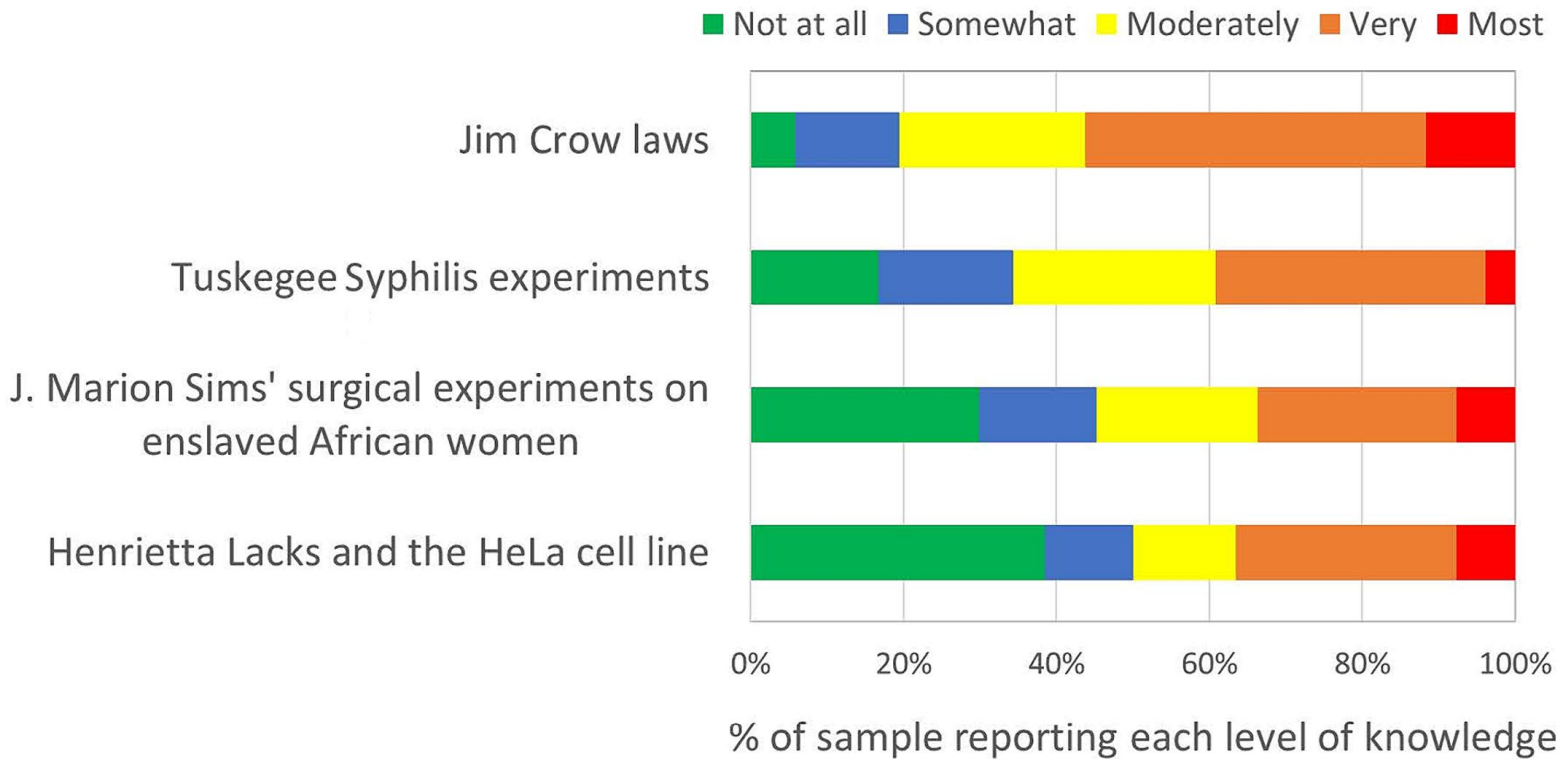
Knowledge of four discrimination-related historical events and policies (*n* = 104) on a scale ranging from “Not at all knowledgeable” to “Most knowledgeable (expert)”.

Figure 1 summarizes the survey respondents’ knowledge of four historical events and policies targeting Black populations. Respondents reported being most knowledgeable about Jim Crow laws and least knowledgeable about Henrietta Lacks and the HeLa cell line (Table 1).

### Respondent characteristics associated with research access and participation

**Figure 2:**
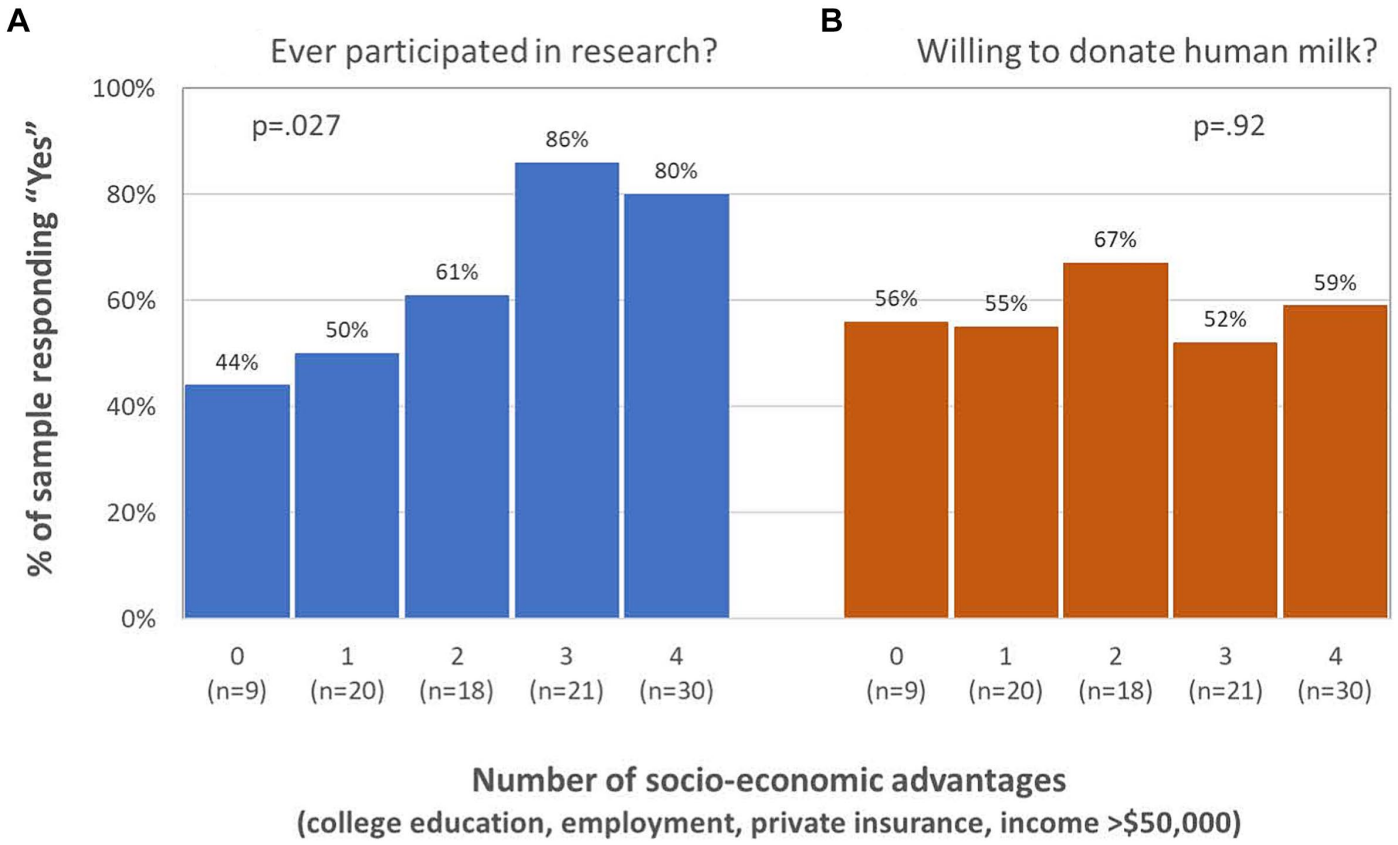
Greater socio-economic advantage is associated with **(A)** higher rates of prior research participation (*p* = 0.027), but not with **(B)** willingness to donate human milk to research (*p* = 0.92).

Prior participation in a research study was significantly associated with a higher socio-economic composite score (*p* = 0.027, see Figure 2). Respondents with at least 3 socio-economic advantages were 4.1 times more likely to have participated in a research study as respondents with fewer than 3 socio-economic advantages (odds ratio = 4.1; 95% CI = 1.6, 10.3; *p* = 0.002). The indicators of socio-economic advantage most strongly associated with prior research participation were having a college degree (78% vs. 43%, *p* = 0.001) and income above $50,000 per year (80% vs. 58%, *p* = 0.016). Being employed or having private insurance were not significantly associated with research participation in this sample.

**Figure 3:**
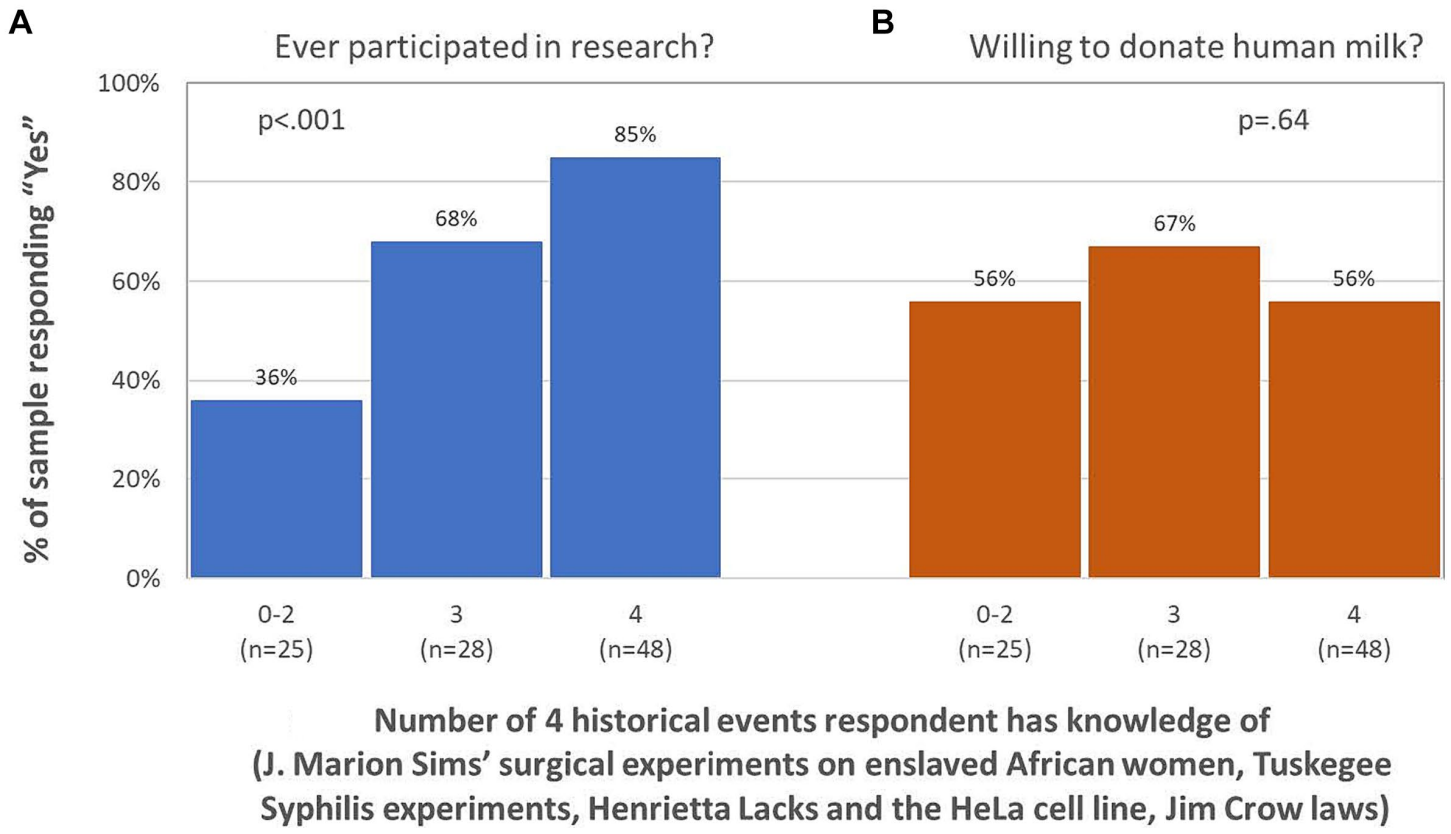
Greater knowledge of Black-targeted historical events/policies is associated with **(A)** higher rates of prior research participation (*p* < .()OI), but not with **(B)** willingness to donate human milk to research (*p* = 64).

Prior participation in a research study was also associated with a higher knowledge composite score (*p* < 0.001, see Figure 3). Compared to respondents who reported knowledge of 0–2 of the historical events and policies, those knowledgeable of 3 were 3.8 times more likely to have participated in prior research (odds ratio = 3.8; 95% CI = 1.2, 11.7; *p* = 0.023) and those knowledgeable of all 4 were 10.4 times more likely to have participated in prior research (odds ratio = 10.4; 95% CI = 3.3, 32.7; *p* < 0.001). These associations were attenuated but remained significant when controlling for education (adjusted odds ratio for knowing 3 events/policies = 3.3, 95% CI = 1.02, 10.6; *p* = 0.046; adjusted odds ratio for knowing 4 events/policies = 7.7, 95% CI = 2.4, 25.4; *p* = 0.001). Knowledge of Henrietta Sacks and the HeLa cell line was the historical event most strongly associated with prior research participation, as respondents reporting knowledge of this event were 9.2 times more likely to have prior research participation than those who reported no knowledge of it (odds ratio = 9.2; 95% CI = 3.6, 23.6; *p* < 0.001; education-adjusted odds ratio = 7.2; 95% CI = 2.7, 19.3; p < 0.001). Prior participation in research was unrelated to respondents’ age, pregnancy status at the time of the survey (pregnant or not), and region of residence.

Unlike prior research participation, willingness to donate human milk to a research study was not associated with respondents’ socio-economic composite score (*p* = 0.92, see Figure 2) nor to any of the 4 indicators of socio-economic advantage. Respondents with no socio-economic advantages were just as likely as those with all 4 advantages to be willing to donate human milk to research (56% vs. 59%). Similarly, willingness to donate human milk to a research study was not associated with respondents’ knowledge composite score (*p* = 0.64, see Figure 3) nor to knowledge of any of the 4 historical events and policies. Respondents with little knowledge of these events and policies were just as likely as those with more knowledge to be willing to donate human milk to research. Willingness to donate human milk was also unrelated to respondents’ pregnancy status and region of residence, but respondents who were willing to donate human milk were significantly younger than those who were unwilling or unsure about donating [30.8 years (SD 4.2) vs. 33.8 years (SD 5.4), *p* = 0.002].

Due to the small numbers of respondents having been asked to participate in a breastfeeding/chestfeeding and/or human milk research study, potential associations between being asked and respondents’ socio-economic advantage and knowledge of historical events could not be statistically evaluated in this sample.

## Discussion

We conducted a national, cross-sectional online survey of Black pregnant and postpartum people and found that a majority (69%) of respondents had participated in some type of research study, prior to being recruited for this study. Yet, very few (8%) of our study participants had ever been recruited to participate in breastfeeding/chestfeeding or human milk research studies. Surprisingly, most of our study participants (59%) shared that they would be willing to donate breast or human milk to a research study, if asked. These findings are important as they challenge current negative narratives related to Black women and biomedical and social science research participation (21). There is an erroneous notion that Black women and birthing people are not interested in research. These assumptions are harmful and further exacerbate existing biases and structural barriers. While issues related to perceptions, mistrust, or historical events do contribute to the lack of diversification noted in biomedical research studies, they are not the only drivers (22–24). Findings from our study suggest that one driver often overlooked may be access. Wendler *et al* noted that willingness to participate in health research differed slightly among racially minoritized and white individuals and that efforts to diversify research participation should focus on increasing access to study opportunities and not necessarily changing attitudes and perceptions (25).

Regarding breastfeeding/chestfeeding and human milk research, Palmquist et al. (17) noted that human milk researchers often do not address the ways systemic, structural, and historical factors – such as racism, colonialism, and power and privilege – impact and/or influence study design, data collection, analysis, and interpretation. Of the few participants in our study who had previously participated in breastfeeding/chestfeeding and human milk research (*n* = 3), only one participant noted that the research team was diverse or consisted of a Person of Color. Further, inequities and disparities noted among the recruitment of diverse study samples in this field may be attributed to the lack of diversity among research teams, limited understanding of root causes of inequities and disparities, and different priorities. Health disparities and inequities are symptomatic of larger societal, historical, and economic policies and practices which perpetuate harm and rooted in structural racism (26). Researchers who share identities with marginalized populations are more likely to pursue research that addresses the needs of these communities, while contributing to the innovation that diverse research teams offer. Moreover, research has shown, minoritized researchers in the health professions are more likely to come from and want to serve historically excluded and minoritized populations and conduct health equity research (27). Strategic measures that address ethical and equitable community-engagement, such as those highlighted by the Breastmilk Ecology: Genesis of Infant Nutrition (BEGIN) Project Working Group (28) and critical investments are needed to address the lack of diversity in the breastfeeding/chestfeeding and human milk research pipeline (17).

Our study also found that prior research participation was associated with greater socio-economic advantage and increased knowledge of historical research activities or events such as the USPHS Syphilis Study at Tuskegee, HeLa cell line, and Marion Sims’ inhumane experimentation of enslaved African women, etc. These findings are consistent with previous literature (29, 30). Henderson et al. (29), which focused on identifying Social Vulnerability Indicators and research participant attrition, found that financial-resource strain was associated with participants’ inability to full participate and engage in their research study. Durant et al. (30) noted that previous clinical trial participation and not race was a higher predictor of future research participation. Additionally, they also found that knowledge of the Tuskegee Syphilis Study was positively associated with Black study participants’ willingness to participate in research (30). This finding along with our data reinforce the importance of knowledge and awareness of past egregious research activities in this population. While interesting, this finding does not negate the lasting impact of mistrust and mistreatment experienced by Black communities (22).

Further, trust was a prominent finding in our study. Regarding research recruitment and engagement about breastfeeding/chestfeeding and human milk research studies, participants overwhelmingly preferred to be engaged in healthcare and/or community settings. These findings align with previous work focused on efforts to increase Black women representation in biomedical research studies (24, 31, 32). Authentic community engagement complimented by cultural humility are critical to the recruitment and retention of Black women and birthing people in breastfeeding/chestfeeding and human milk research studies. In addition, trusted healthcare professionals and individuals, such as Lactation Support Providers (e.g., International Board-Certified Lactation Consultants, Certified Lactation Educators, Certified Lactation Counselors, and Breastfeeding Peer Counselors) and Doulas were identified as the most preferred individuals to discuss breastfeeding/chestfeeding and human milk research studies with Black pregnant and postpartum people in our study, followed by Healthcare Providers. This finding is important as Lactation Support Providers, especially International Board-Certified Lactation Consultants (IBCLCs) can serve as a bridge between research teams and potential participants. IBCLCs have a solid understanding of human milk feeding and lactation and are required to complete an Introduction to Clinical Research course or similar content in order to obtain and/or renew their certification (33, 34). Given that Lactation Support Providers, along with Doulas, are found in both acute care and community-settings, collaborating with these professions may be an important strategy to increasing diversity among breastfeeding/chestfeeding and human milk research study participants.

### Limitations

The perspectives presented in this article were from a small sample of Black pregnant and postpartum women. There is a risk of sampling bias, as this convenience sample of women who responded to study flyers and advertisements may differ from the broader population of Black pregnant and postpartum people, particularly those who do not identify as women. Due to the size of the sample, the confidence intervals for the reported associations are large, and small cell sizes precluded some analyses. Future research with a larger sample and/or including qualitative methods is needed to replicate and further elucidate these findings.

## Conclusion

Inequities and disparities related to biomedical research participation persist. Thus, it is important to understand barriers and challenges associated with human milk research participation among Black pregnant and postpartum people. Limited discussions are being had in the human milk feeding and lactation landscape regarding increasing diversity among research participants. Greater efforts are needed to increase access to research opportunities, diversity research teams, and build collaborations with trusted community partners. A lack of diversity in human milk research is a missed opportunity for scientific discovery. Additional research is needed to develop recruitment and retention solutions, which are responsive to community concerns and reflect guidance from the communities most impacted by health inequities and disparities.

## Data availability statement

The raw data supporting the conclusions of this article will be made available by the authors, without undue reservation.

## Ethics statement

This study was approved by the UCSF Institutional Review Board and conducted in accordance with the local legislation and institutional requirements. The study participants provided both verbal consent during the eligibility screening and online consent as part of the online survey.

## Author contributions

IA: Conceptualization, Funding acquisition, Writing – original draft, Writing – review & editing, Project administration. CG: Formal analysis, Writing – original draft, Writing – review & editing. BG-B: Writing – review & editing. GN: Project administration, Writing – review & editing.
